# TRIPLE MALADY: OLDER PERSONS WITH MENTAL ILLNESSES AND COVID- 19: INSIGHTS FROM A PSYCHOGERIATRIC CLINIC IN NIGERIA

**DOI:** 10.1093/geroni/igac059.1804

**Published:** 2022-12-20

**Authors:** Olubukola Omobowale, Olufisayo Elugbadebo, Oluwagbemiga Oyinlola

**Affiliations:** University of Ibadan, Nigeria, Ibadan, Oyo, Nigeria; College of Medicine, University of Ibadan, Ibadan, Oyo, Nigeria; University College Hospital, Ibadan, Ibadan, Oyo, Nigeria

## Abstract

**Background:**

Older persons with mental illnesses have been differentially affected by COVID-19 because of reduced access to routine health care, the adverse social impacts of the preventive strategies, and inadequate knowledge of the ongoing COVID -19 pandemic.Adequate knowledge is crucial to ensuring adherence to the right preventive practices among older persons.

**Methods:**

A hospital-based comparative study was conducted among older persons attending the Psychogeriatric and Healthy Ageing clinics of the Geriatric Centre, University College Hospital, Ibadan. Data were gathered with a semi-structured interviewer administered questionnaire and SPSS version 23 was used to analyze the data. The level of significance was set at 5%.

**Results:**

A total of 390 respondents aged 60 and above were sampled in the two groups: 195 with a psychiatric diagnosis (PD) and 195 with a non-psychiatric diagnosis (nPD). Their mean age was PD: 72.2 (±7.4) years and nPD: 71.0 (±8.0) years. About 14.9% had dementia and mild cognitive impairment; 14.6% psychotic disorders; 13.8% mood disorders and 6.6% anxiety and somatoform disorders. The majority were aware of the ongoing pandemic (PD: 95.9%; nPD: 96.4%). The use of facemask (PD: 89.7%; nPD: 86.7%) was the commonest preventive practice. Male gender (OR:2.09, CI; 1.14-3.86, p= 0.018) and education (OR: 5.10, CI; 1.15-22.67, p= 0.032) were predictors among PD and nPD respectively.

**Conclusion:**

Older persons with psychiatric diagnoses have gaps in their knowledge of COVID-19 compared to those without symptoms. Health education about the ongoing pandemic and prevention programs targeting the older population with mental illnesses would be beneficial.

